# Laser-Induced Nanowire Percolation Interlocking for Ultrarobust Soft Electronics

**DOI:** 10.1007/s40820-024-01627-7

**Published:** 2025-01-31

**Authors:** Yeongju Jung, Kyung Rok Pyun, Sejong Yu, Jiyong Ahn, Jinsol Kim, Jung Jae Park, Min Jae Lee, Byunghong Lee, Daeyeon Won, Junhyuk Bang, Seung Hwan Ko

**Affiliations:** 1 Applied Nano and Thermal Science Lab, Department of Mechanical Engineering, Seoul National University, 1 Gwanak-ro, Gwanak-gu, Seoul, 08826 South Korea; 2 Energy Device Research Team, Hyundai Motor Company, 37 Cheoldobangmulgwan-ro, Uiwang-si, Gyeonggi-do 16082 South Korea; 3 Institute of Advanced Machinery and Design (SNU-IAMD), Seoul National University, Gwanak-ro, Gwanak-gu, Seoul, 08826 South Korea

**Keywords:** Nanowire percolation network, Laser processing, Mechanical interlocking, Functionalization, Conducting polymer

## Abstract

Metallic nanowires have served as novel materials for soft electronics due to their outstanding mechanical compliance and electrical properties. However, weak adhesion and low mechanical robustness of nanowire networks to substrates significantly undermine their reliability, necessitating the use of an insulating protective layer, which greatly limits their utility. Herein, we present a versatile and generalized laser-based process that simultaneously achieves strong adhesion and mechanical robustness of nanowire networks on diverse substrates without the need for a protective layer. In this method, the laser-induced photothermal energy at the interface between the nanowire network and the substrate facilitates the interpenetration of the nanowire network and the polymer matrix, resulting in mechanical interlocking through percolation. This mechanism is broadly applicable across different metallic nanowires and thermoplastic substrates, significantly enhancing its universality in diverse applications. Thereby, we demonstrated the mechanical robustness of nanowires in reusable wearable physiological sensors on the skin without compromising the performance of the sensor. Furthermore, enhanced robustness and electrical conductivity by the laser-induced interlocking enables a stable functionalization of conducting polymers in a wet environment, broadening its application into various electrochemical devices.

**Supplementary Information:**

The online version contains supplementary material available at 10.1007/s40820-024-01627-7.

## Highlights

- Laser-induced percolation interlocking technology enables the development of the robust, open-structured nanowire (NW) electrodes through the photothermal effect at the interface between NW and substrate.
- The optimized NW electrode with enhanced mechanical and electrical properties can be used as reusable wearable electronics.
- Stable functionalization of the percolation-interlocked NW electrode with various conducting polymers can be achieved, broadening the applicability as soft electronics.

**Supplementary Information:**

The online version contains supplementary material available at 10.1007/s40820-024-01627-7.

## Graphical abstract

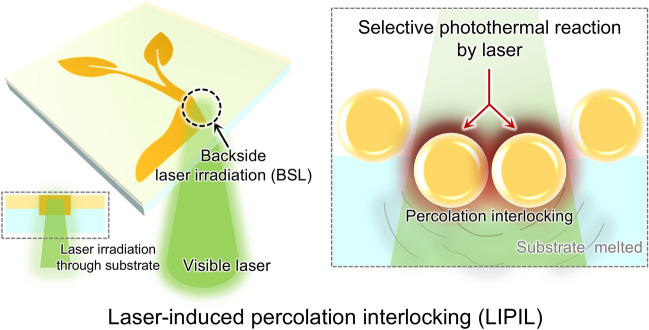

## Introduction

Metallic nanowires (NWs) are recognized as exceptional electrical conductors for soft electronics due to their high electrical conductivity and stretchability [1–3]. However, the NW networks inevitably require a supportive substrate, since they cannot remain freestanding [4]. In this context, NW-based conductive fiber composites have achieved freestanding structures with high stretchability but remain vulnerable to friction-induced shear stress [5–7]. For sustainable functionality of the NW network, it is crucial to ensure the stability and integrity of the assembled structure comprising the NW electrode and the substrate under continuous external stimuli [8]. Because of the mechanical fragility of the NW network on the substrate, it has been encapsulated with protective layers to preserve its structure for its stable operation under external stimuli [9–11]. However, the presence of a protective layer prevents the NW network from directly interacting with surrounding environments, significantly restricting its potential for advanced applications, such as neutral probes and physiological sensors that require direct contact with biological systems [12–15]. In this regard, the NW network should be utilized as an open structure with high robustness to enhance its interaction capabilities.

Furthermore, an exposed structure of NW networks can further maximize the potential of NW networks, enabling the direct introduction of conducting polymers (CPs) on NW surfaces [16–18], which impart new functionalities with electrochemical interactions with surrounding environments [19, 20]. The high surface area of CP layers provided by the NW network enhances electrochemical interactions [21], making them suitable for various applications, including energy-harvesting devices [22], energy storage devices [23, 24], and smart windows [25, 26]. However, particularly in NW-based electrodes, the general solution-based electropolymerization (EP) process for depositing CPs directly onto the electrode leads to the dispersion of NWs into the solution, ultimately causing their failure due to weak adhesion with the substrate. Therefore, a robust open structure is necessary to ensure the stability and functionality of NW networks for electrochemical applications.

In this respect, laser-based thermal interactions such as sintering [27], reduction [28], and pyrolysis [29] have played a crucial role in fabrication of nanomaterial-based electrodes for soft electronics. Moreover, selective laser-based interactions have facilitated the rapid patterning of functional electrodes and the precise tailoring of their properties for specific applications [30]. However, the limited photothermal reaction in the depth direction hindered effective interactions between the electrode nanomaterials and the substrate for robust open structure of the electrode. As a result, the weak adhesion and mechanical mismatch between the electrodes and the substrate presented significant challenges in achieving the strong bonding necessary for practical applications [31, 32].

Herein, we present a versatile laser-induced percolation interlocking (LIPIL) method to achieve a robust open NW structure on a substrate, applicable to various metallic NWs as well as diverse substrates. Unlike conventional laser-based techniques, we irradiated laser onto the NW percolation network from the backside of the supporting substrate to concentrate the photothermal energy at the interface between the NW electrode and substrate. Consequently, the backside laser (BSL) irradiation embedded the NW network into the polymeric substrate, resulting in a highly durable open structure of NW network which exhibited exceptional mechanical stability under severe cyclic mechanical tests even without a protective layer. In comparison with existing embedding methods that degrade NWs, our LIPIL technique offers a more efficient solution for directly embedding NWs into substrates without causing any degradation (Supplementary S1). Furthermore, a selective LIPIL method facilitates high-resolution direct patterning of NWs, enabling the development of long-term reusable electrophysiological sensors. Furthermore, our approach allows the NW network to stably maintain its percolation network during the solution-based EP process, which is crucial for the functionalization of NW electrode with CPs. Finally, we demonstrated the effectiveness of LIPIL in maximizing the potential of NWs by decorating with various CPs to fabricate electrochemical devices, including visible (VIS)-to-infrared (IR) electrochromic (EC) devices, supercapacitors, and biosensors. Therefore, this study will drive the development of more advanced technologies that surpass the current level of nano-micro-fabrication technology [33–35].

## Experimental Section

### Synthesis of Cu NWs

Cu NWs were synthesized via a hydrothermal method using copper (II) chloride dihydrate (CuCl_2_·2H_2_O, Sigma-Aldrich) as the precursor, hexadecylamine (HDA, Sigma-Aldrich) as the ligand, and glucose (Sigma-Aldrich) as the reducing agent. Initially, 0.84 g of CuCl_2_·2H_2_O and 5.2 g of HDA were thoroughly dissolved in 400 mL of deionized (DI) water with continuous stirring. Subsequently, 2 g of glucose was added to the mixture, and the solution was heated to 100 °C for 7 h 20 min. As the NWs formed, the solution gradually changed to a reddish-brown color. Finally, the Cu NW solution was purified by centrifuging at 1500 rpm for 15 min, using isopropanol (IPA, SAMCHUN Chemicals) multiple times. The prepared Cu NWs solution was adjusted to possess a concentration of 10.5 mg mL^−1^.

### Synthesis of Ag NWs

Ag NWs were synthesized using a polyol-mediated process, adapted from previous research to increase the length of the NWs [36]. Initially, 3.182 g of polyvinylpyrrolidone (PVP, M_w_ = 360,000, Sigma-Aldrich) were dissolved in 260 mL of ethylene glycol (EG, SAMCHUN Chemicals) and preheated to 175 °C while being stirred at 130 rpm. Once the temperature stabilized, 1.6 mL of a 4 mM copper (II) chloride dihydrate (CuCl_2_·2H_2_O, Sigma-Aldrich) solution was added. Then, 60 mL of a 100 mM silver nitrate (AgNO_3_, SAMCHUN Chemicals) solution in EG was gradually injected at a rate of 3 mL min^-1^ using a syringe pump. After the injection was completed, stirring was stopped, and the reaction mixture was left undisturbed for 2 to 3 h. The resulting Ag NWs were diluted with 900 mL of acetone (SAMCHUN Chemicals), and the residual solution was removed to collect aggregated NWs. These aggregates were dispersed in distilled water and washed multiple times by centrifugation at 2500 rpm for 5 min each time. Finally, the purified Ag NWs solution was adjusted to possess a concentration of 16 mg mL^−1^.

### Synthesis of Ag-Au (AA) Core–Shell NWs

AA NWs were synthesized by modifying methods from a previous study [37]. First, an Au growth solution was prepared by dissolving 30 mg of chloroauric acid trihydrate (HAuCl_4_·3H_2_O, Sigma-Aldrich), 33 mg of sodium hydroxide (NaOH, Sigma-Aldrich), and 17 mg of sodium sulfite (Na_2_SO_3_, Sigma-Aldrich) in 70 mL of DI water. This solution was kept at 5 °C for 12 h. Concurrently, another solution was prepared by combining 800 mg of PVP (M_w_ = 40,000, Sigma-Aldrich), 70 mg of NaOH, 300 mg of L-ascorbic acid (L-AA, SAMCHUN Chemicals), and 10 mg of Na_2_SO_3_ in 100 mL of the Ag NW solution. The Au growth solution was then gently mixed with the Ag NW solution. After 60 min of mixing, the Au shell was successfully formed around the Ag NW core. Finally, the synthesized core–shell NWs were purified through several rounds of centrifugation and then dispersed in ethanol for further use. The concentration of the purified AA NWs solution was set to 18.2 mg mL^−1^.

### Synthesis of Ag-(Au-Pt) (AAP) Core–Shell NWs

The AAP NWs were synthesized using a method similar to that used for AA NWs [38]. To prepare the (Au–Pt) growth solution, 140 mg of HAuCl_4_·3H_2_O, 110 mg of NaOH, and 132 mg of Na_2_SO_3_ were mixed in 40 mL of DI water. Following this, 70 mg of chloroplatinic acid hexahydrate (H_2_PtCl_6_·nH_2_O, Sigma-Aldrich) was added to the mixture. The solution was then diluted with 240 mL of DI water and left undisturbed at 5 °C for 24 h. Separately, another solution was prepared by mixing 3.6 g of PVP (M_w_ = 40,000), 280 mg of NaOH, 1.32 g of L-AA, 44 mg of Na_2_SO_3_, and 15 mL of Ag NWs solution in 395 mL of DI water. After preparing these solutions, the (Au–Pt) growth solution was carefully combined with the Ag NWs solution. After 120 min, the (Au–Pt) alloy shell had successfully formed around the Ag NWs core. The resulting core–shell NWs were then washed multiple times through centrifugation and dispersed in ethanol. Finally, we redispersed the AAP NWs in ethanol to obtain a concentration of 13.3 mg mL^−1^.

### Preparation of Bare, Welded, Percolation-Interlocked (PIL) NW Electrode

For the preparation of the bare sample, the target NWs were deposited using a vacuum filtration process on the target substrate. Then, by utilizing the continuous wave 532 nm wavelength laser (Sprout-G-5W, Lighthouse Photonics), the welded and PIL NW electrodes were prepared. Specifically, for the preparation of the welded sample, the bare NW electrode sample was placed on the stage of the laser setup with the NW surface facing upward. Then, the laser was directly irradiated onto the NW surface. Meanwhile, for the preparation of the PIL NW electrode, the bare NW electrode sample was placed on the stage of the laser setup with the transparent substrate facing upward (the NW surface facing downward), and then, the laser was directed through a transparent substrate, followed by irradiation of the NW surface.

### Material Characterization

The Scanning electron microscope (SEM) images were acquired using field emission (FE)-SEM (JSM-7600, JEOL). The transmission electron microscope (TEM) images were obtained using FE-TEM (Tecnai F20, FEI). The atomic force microscopy (AFM) images were acquired by scanning the surfaces of bare, welded, and PIL samples (NX-10, Park Systems). The sheet resistance was assessed by using a 4-point probe (2002 multimeter, Keithley). The electrical resistances were measured using a two-point probe method for the sonication-resistant test and crumpling test (DMM7510, Keithley). A laboratory-scale setup comprising Keithley apparatus and linear stages was utilized to measure the resistance change of the electrode samples for the characterization of stretching, bending, and friction tests. The thermal images were obtained using an IR camera (FLIR A645 sc). The Fourier transform infrared (FTIR) data were obtained by FTIR spectroscopy (Spectrum Paragon, PerkinElmer). The IR emissivity data were measured by FTIR spectroscopy (Nicolet iS50 FTIR, Thermo Scientific) equipped with an integrating sphere (mid-IR integratIR, Pike Technologies). The electrochemical properties, including skin impedance, cyclic voltammetry (CV) data, galvanic charging and discharging data, and differential pulse voltammetry (DPV) data, were measured using an electrochemical workstation (VersaSTAT 3, Princeton Applied Research).

### Measurement of Electromyography (EMG) Signals

For measuring the EMG signals, the working, reference, and counter electrodes were fabricated using a laser-selective patterning process of AA NWs. Then, the as-prepared electrodes were attached to the musculocutaneous region of the human forearm, followed by measuring the EMG signals with an EMG acquisition module (BioRadio, Great Lakes NeuroTechnologies).

### Fabrication of Functionalized NW-Based Device Using Various CPs

To fabricate the functionalized NW-based devices using various CPs, the EP was conducted using the electrochemical workstation with a three-electrode method, consisting of PIL AA NWs as the working electrode, a Pt mesh electrode as the counter electrode, and an Ag–AgCl electrode as the reference electrode.

For an EC device using polyaniline (PANI), the PIL AA NWs were immersed in a solution containing 1 M perchloric acid (HClO_4_, SAMCHUN Chemicals) and 0.5 M aniline (SAMCHUN Chemicals). The EP was then performed at a steady current density of 0.2 mA cm^−2^. Following this process, the electropolymerized PANI films were thoroughly rinsed with DI water.

For a supercapacitor using polypyrrole (PPy), the PIL AA NW electrode was submerged in a solution of 1.0 M potassium nitrate (KNO_3_, SAMCHUN Chemicals) and 0.1 M pyrrole (Sigma-Aldrich). The EP of PPy was performed through a cyclic potential sweep from 0 to 1.0 V at a scan rate of 0.2 V s^−1^ for 20 cycles. Finally, the composite film was washed with DI water, and dried at room temperature. Then, the gel electrolyte was prepared by mixing 6 g polyvinyl alcohol (PVA, M_w_ = 89,000–98,000, Sigma-Aldrich) with 6 g of phosphoric acid (H_3_PO_4_, Sigma-Aldrich) in 60 g of DI water at 85 °C. Then, the AA-PPy core–shell NW films were immersed in the prepared gel electrolyte for 1 min and dried for 4 h. During the drying process, the gel electrolyte was changed to a sticky thin film.

For a molecularly imprinted polymer (MIP)based biosensor using poly(3,4-ethylenedioxythiophene)-poly(styrenesulfonate) (PEDOT:PSS), the PIL AA NW electrode was immersed in a solution containing 0.01 M 3,4-ethylenedioxythiophene (EDOT, Sigma-Aldrich), 0.1 M poly(sodium 4-styrenesulfonate) (NaPSS, Sigma-Aldrich) and 1 M urea (Sigma-Aldrich). Then, the EP time was set to 30 s at a constant potential of 1 V, as it exhibited the highest sensitivity. After electropolymerization, the urea-embedded MIP-based sensor was eluted by submerging the as-fabricated sensor into the DI water for an hour, followed by drying at room temperature.

## Results and Discussion

### Laser-Induced NW Percolation Network Interlocking Within Substrates

**Fig. 1:**
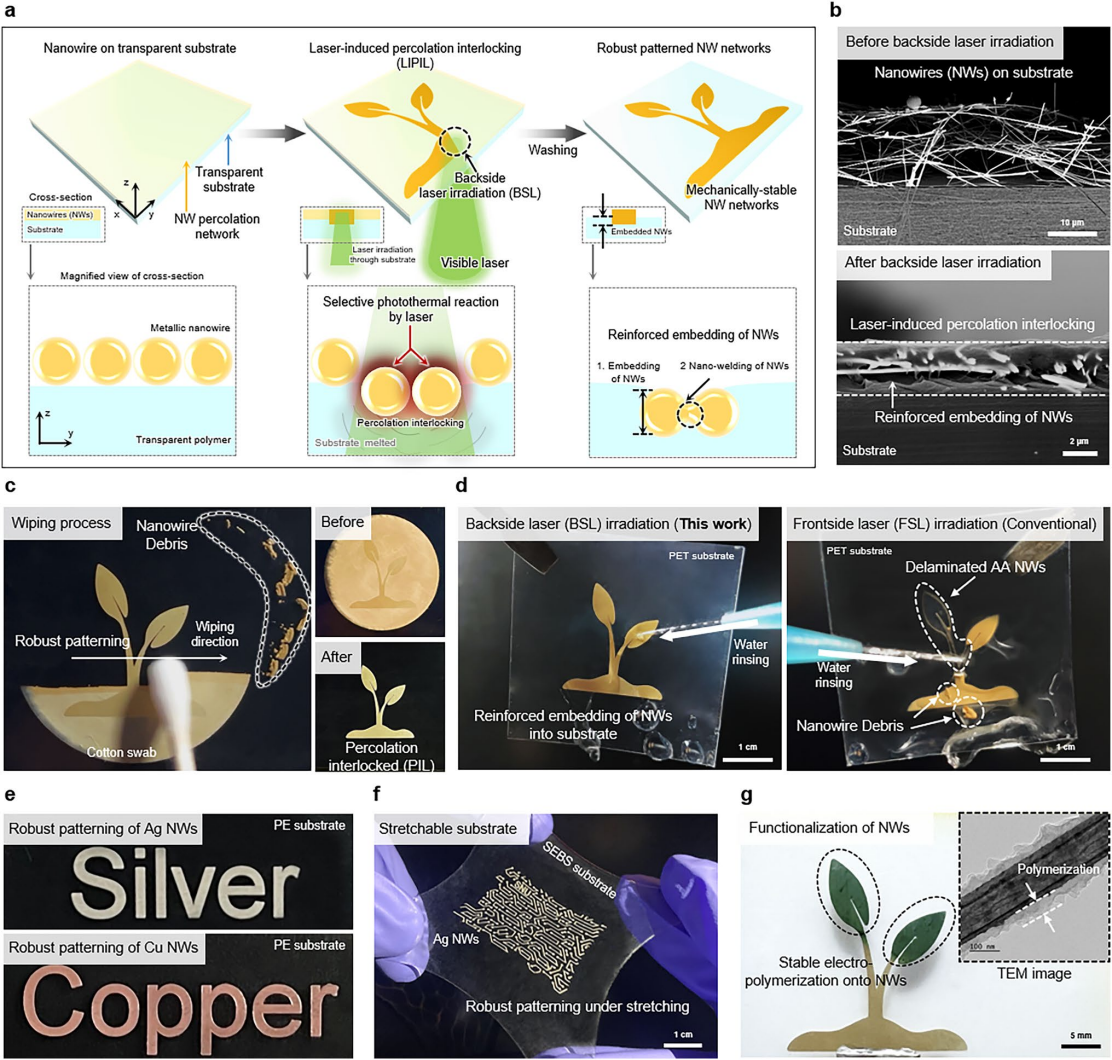
LIPIL process of NWs within the substrates. **a** Schematic illustration of LIPIL. **b** SEM images of the cross-section of vacuum-filtrated NWs on the substrate (top) and PIL NWs by laser (bottom). **c** PIL NWs by laser, showing the high robustness even when subjected to strong external stimulus such as wiping process with cotton swab. **d** Comparison of NWs fabricated by our method (left) and conventional method (right), showing the robust patterning of PIL NWs on the substrate. **e** Patterned PIL Ag NWs (top) and Cu NWs (bottom) on the PE substrate. **f** Stretchable LED circuit using patterned PIL Ag NWs on the SEBS substrate. **g** Functionalization of AA NWs with PANI. The inset shows the TEM image of the functionalized NW.

To begin, the NWs were vacuum-transferred onto a transparent thermoplastic substrate to establish an NW percolation network (Fig. 1a). To achieve a robust, open structure of NW networks on the substrate, we irradiated a 532 nm continuous wave laser (laser beam diameter ~ 17.55 μm) through the backside of the substrate (Fig. 1a, middle, and Fig. S1). The laser beam is transmitted through the transparent substrate, concentrating the photothermal energy at the interfacial layer between the NW electrode and the substrate. The concentrated photothermal reaction induced by surface plasmonic resonance causes the transient melting of the substrate, resulting in the interlocking of the NW percolation network through the interpenetration of NWs and the polymer matrix (Fig. 1a, right). Consequently, the selectively embedded NW network remains intact even though it is wiped due to enhanced mechanical stability. Furthermore, this embedded NW network fabricated by the LIPIL process exhibits improved electrical properties due to the nano-welding of NWs at the junction [39, 40]. In contrast to the conventional frontside laser (FSL) irradiation, which directly targets the NW surface and induces only NW welding, the LIPIL process surpasses the FSL method by enabling both effective NW welding and simultaneous embedding into the substrate. In this study, the ‘reinforced’ NWs refer to the nano-welded NWs that are simultaneously embedded within the substrate, thereby exhibiting enhanced mechanical and electrical properties. SEM images confirmed that the reinforced AA NWs by the LIPIL method were successfully embedded within the substrate while being exposed to the surrounding environments as an open structure (Fig. 1b). With the selective LIPIL processing, the PIL NW network can be easily patterned into the desired electrode shape, enduring external stimuli such as cotton swab wiping (Fig. 1c). The PIL NW electrode by BSL irradiation can withstand the water pressure during rinsing, whereas the NW electrode by FSL irradiation delaminates under such conditions (Fig. 1d).

Most importantly, the LIPIL method is universally applicable to various metallic NWs and transparent thermoplastic substrates, allowing for the tailored selection of NWs to meet specific application requirements. For instance, Ag NWs, preferred for their high electrical conductivity, and Cu NWs, favored for their cost-effectiveness, can both be selectively patterned using our LIPIL method (Figs. 1e and S2). In addition, the LIPIL method is effectively applied to various transparent substrates, including polyethylene (PE), polyethylene terephthalate (PET) film, thermoplastic polyurethane (TPU) and styrene-ethylene-butylene-styrene (SEBS) films for developing the flexible and stretchable electronics (Figs. 1f and S3, S4, Supplementary S2). Lastly, the robust, open structure of NWs enabled by the LIPIL process facilitates direct deposition of CPs onto the NW surface using a solution-based EP process, enhancing the functionality of NW electrodes with electrochemical reactions. For example, we could selectively deposit PANI onto PIL AA NWs, without causing dispersion of NWs in the solution during the EP process (Fig. 1g).

### Mechanism of the LIPIL Process and Characterization of the PIL NW Electrodes

The fundamental mechanism of the LIPIL is primarily ascribed to the photothermal effect resulting from laser absorption. Coinage metallic NWs including Au, Ag, and Cu NWs exhibit high absorptivity in the 300–600 nm wavelength range [41]. This strong absorption arises from surface plasmon resonance, which becomes more pronounced when these metals are fabricated at the nanoscale [42]. In metallic NW percolation networks, the plasmonic effect is significantly enhanced at NW junctions, where localized surface plasmon resonance induces intense localized heating [40, 43]. This heating drives nano-welding, facilitating material fusion and the expansion of contact areas. Furthermore, it effectively removes electrically insulating capping agents or surface oxides. These morphological transformations collectively reduce junction contact resistance and enhance electron transport pathways, thereby improving the overall electrical performance of the network. Therefore, we investigated whether the absorptive spectrum induces sufficient photothermal reaction depending on the amounts of NWs (Supplementary S3 and Fig. S5). Furthermore, as electrical conductivity is a critical property of the electrode, we examined the conductivity of the AA NW electrode following irradiation under various laser parameters (Fig. S6). Based on this preliminary parametric study, we fixed the NW volume at 1000 μL to fabricate the electrode with an area of 10.75 cm^2^. Furthermore, based on this study, the laser power was set to 100 mW (laser power density 0.413 mW cm^−2^) for FSL irradiation and 300 mW (laser power density 1.240 mW cm^−2^) for BSL irradiation, with a scan speed of 100 mm s^−1^ for both, which resulted in enhanced electrical and mechanical properties. FSL irradiation requires relatively low power, as the photothermal effect is confined solely to the NW electrode, enabling nano-welding without interaction with the substrate. In contrast, the BSL irradiation requires higher power to compensate for laser attenuation through the substrate, facilitating interlocking via transient substrate melting and enhancing both electrical and mechanical stability.

**Fig. 2:**
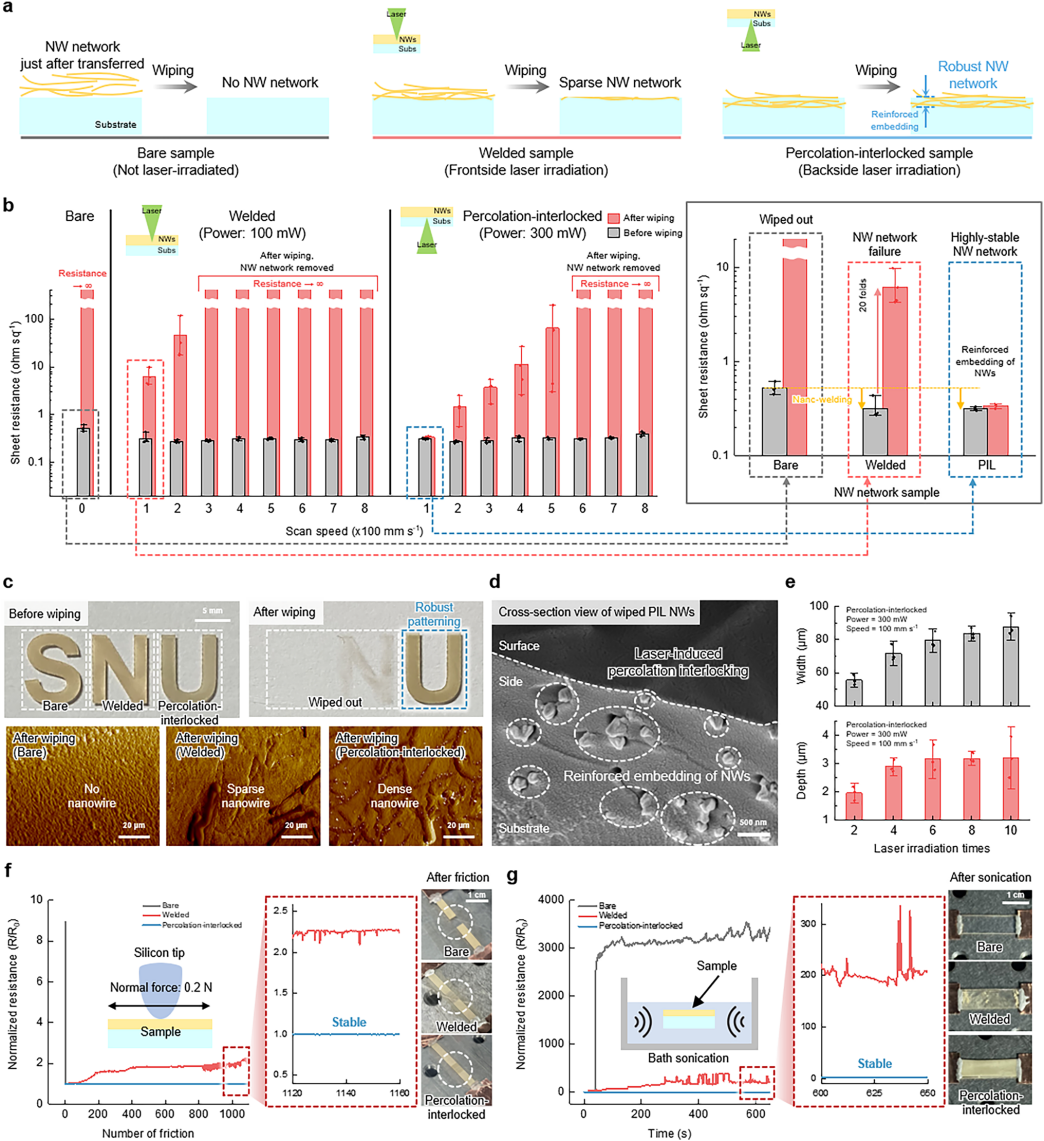
Characterization for NW electrodes produced by LIPIL process. **a** Schematic illustration of three types of samples, which are subjected to the wiping process. Bare sample: NWs just after transferred onto the substrate using vacuum filtration method. Welded sample: NWs fabricated by the FSL irradiation onto the NW surface. PIL sample: NWs fabricated by the BSL irradiation through the substrate onto the interface between NWs and substrate. **b** Sheet resistance of the bare, welded, and PIL samples with varying laser process conditions before and after the wiping process. The inset provides a magnified view of the electrical changes after the wiping process in electrodes under optimized laser conditions, highlighting the differences in mechanical robustness among bare (gray), welded (red), and PIL (blue) electrodes. **c** Digital images (top) and AFM images (bottom), showing the dense NW only in the PIL sample even after the wiping process. **d** SEM image of the cross-section of wiped PIL sample. **e** Patterned width (top) and depth (bottom) dimensions according to laser irradiation times at 300 mW and 100 mm s.^−1^. **f**, **g** Normalized resistance behaviors of the three types of samples during (**f**) the friction test and (**g**) the ultrasonication test. The insets with red dashed lines in Fig. 2f, g provide magnified views of the normalized resistance of welded (red) and PIL (blue) electrodes, highlighting the superior mechanical robustness of the PIL electrode. Data in **b** and **e** represent means ± standard deviation (*n* = 4 and 3, respectively)

To investigate the photothermal effects of laser irradiation in detail, we fabricated three types of NW electrodes: bare, welded, and PIL NW electrodes (Fig. 2a). The bare electrode represents an NW electrode that has been directly vacuum-transferred to the substrate, without any further treatment. The welded electrode is produced by applying FSL irradiation directly onto the NW surface, while the PIL electrode is created with BSL irradiation through the backside of the substrate. When three as-prepared electrodes were subjected to the wiping process, the bare and welded NW-based electrodes were clearly wiped out (Supplementary Video S1). Under FSL irradiation, the laser-induced photothermal effect is primarily confined to the top electrode, causing the heat to spread laterally across the NW electrode (Supplementary S4 and Fig. S7). The photothermal effect limited to the electrode effectively enhances the electrical and mechanical properties of the NW electrode itself. However, the presence of the top electrode restricts the laser penetration depth, thereby impeding the subsequent photothermal effect at the electrode-substrate interface, which is essential for effective NW percolation interlocking and resistance to shear stress. However, the PIL NW-based electrode maintains its robust structure even after the wiping process, demonstrating enhanced stability due to the reinforced embedding of NWs within the substrate (Supplementary Video S2). In the case of BSL irradiation, the laser can transmit through the transparent substrate without significant attenuation by the top electrode, reaching the electrode-substrate interface. This enables a highly localized and concentrated photothermal effect, promoting transient melting of the underlying substrate and facilitating interpenetration between the NWs and the polymer matrix (Fig. S8). As a result, a reinforced PIL NW electrode with improved stability and adhesion is achieved, and it also exhibited superior electrical properties compared to other NW-based electrodes (Table S1).

To experimentally demonstrate the enhanced mechanical stability attributed to the LIPIL effect, we investigated the electrical resistance of each electrode after applying shear stress through a wiping process (Fig. 2b). As expected, the bare electrode was clearly erased just after the wiping process, showing infinite sheet resistance. For the welded electrode, the sheet resistance increased by approximately 20-fold after the wiping process under optimized FSL irradiation. Furthermore, the increase in sheet resistance became more pronounced with higher scan speeds due to the corresponding decrease in laser fluence. In contrast, the PIL electrode showed negligible sheet resistance change due to the reinforced embedding that involves both nano-welding and embedding behavior even after the wiping process. However, like FSL irradiation, BSL irradiation exhibited an increase in sheet resistance as the scan speed increased. Nevertheless, BSL irradiation demonstrated a much more effective interlocking capability compared to FSL irradiation.

We visually examined the LIPIL effect on the patterned NW electrode using the PE substrate. The bare electrode (patterned in the shape of the letter ‘S’) was completely removed after the wiping process. While the welded electrode (patterned as the letter ‘N’) was nearly erased, the PIL electrode (patterned as the letter ‘U’) was preserved without any damage (Fig. 2c). Moreover, the difference in the surface morphology between the samples after the wiping process is obvious in the AFM images. These images demonstrate that the dense NW networks still remain intact in the PIL samples, in contrast to the bare and welded samples. The cross-sectional SEM image confirms that the PIL NWs subjected to the wiping process are securely embedded in the substrate, ensuring high robustness (Fig. 2d).

In addition, the LIPIL effect by BSL irradiation enables not only highly robust structures, but also high-resolution patterning of NW networks through selective irradiation. During a double-scan laser process, the LIPIL effect demonstrated a patterning resolution of 55.56 μm in width and 1.95 μm in depth. As the number of laser scans increased, the patterning resolution saturated at ~ 90 μm in width and ~ 3 μm in depth due to the limited photothermal effect (Fig. 2e). We also confirmed that it is possible to achieve much higher resolution patterning by adjusting the laser parameters (Supplementary S5 and Fig. S9).

To further evaluate the robustness of the PIL NWs for practical applications, we conducted multiple mechanical cyclic tests to assess their stability against external stimuli, simulating real-world scenarios. First, under repetitive friction with a normal force of 0.2 N applied by a silicon tip, the PIL NWs exhibited negligible change in resistance, showing only a variation of 0.01 after 1000 cycles of friction tests, in contrast to the bare and welded NWs (Fig. 2f). Moreover, we confirmed that the PIL samples maintained high electrical properties even after 10 min of ultrasonication, with a minimal resistance variation of 0.25, compared to the bare and welded samples which exhibited much larger variations of 3332.96 and 213.41, respectively (Fig. 2g). The PIL samples on the diverse substrates such as TPU, PET, and SEBS film also exhibited a stable appearance after the ultrasonication process (Fig. S10). Additionally, the PIL NWs exhibited stable endurance against various mechanical stresses such as bending, stretching, and even crumpling (Figs. S11–S13). Lastly, our LIPIL method is highly suitable for soft electronics because it does not cause substrate deformation due to localized heating unlike bulk annealing that results in plastic deformation of the substrate (Fig. S14).

### Physiological Signal Monitoring Application with LIPIL NW Electrode

**Fig. 3:**
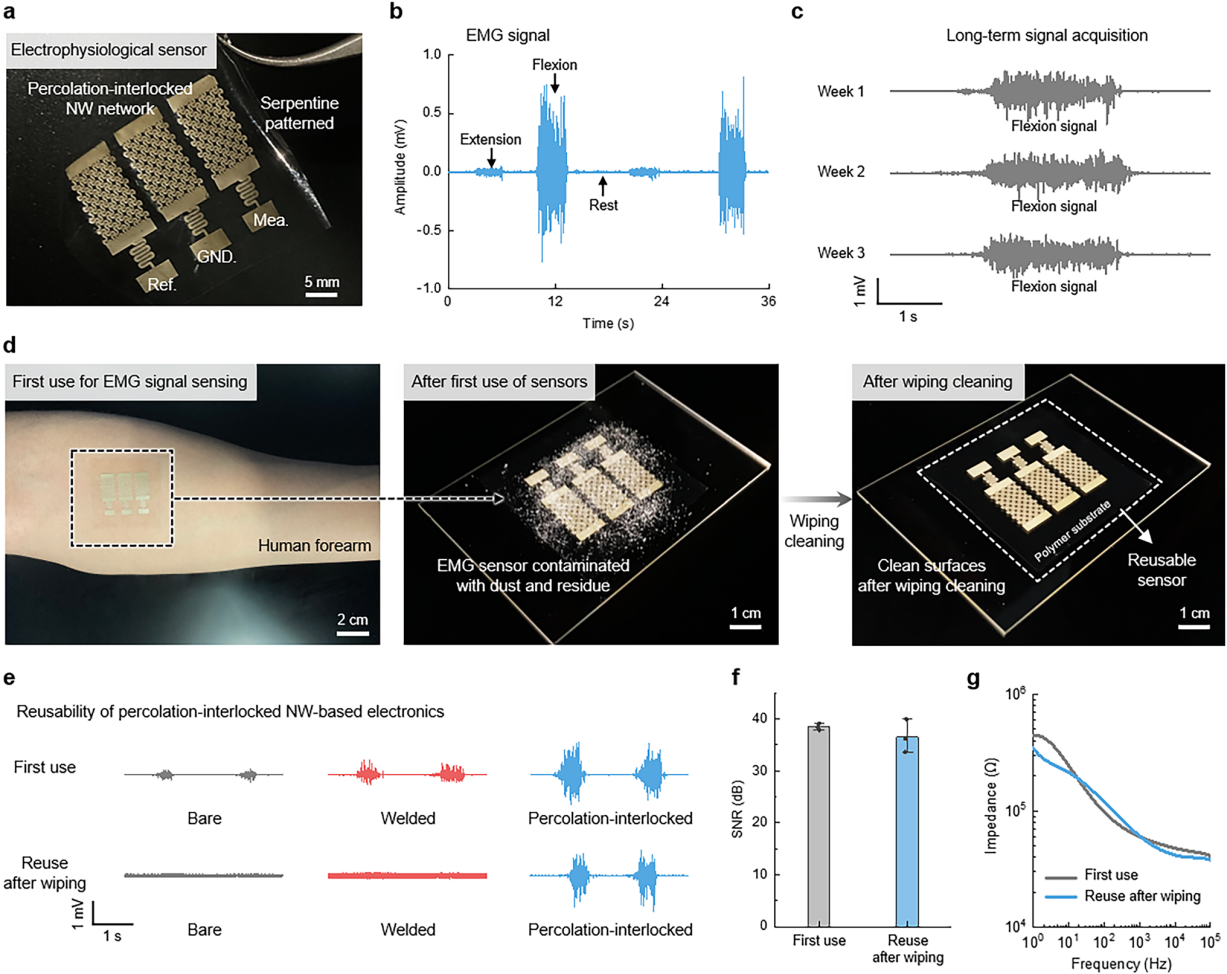
Electrophysiological sensing applications. **a** Digital image of laser-patterned PIL NWs for sensing the EMG signals. **b** EMG signal acquisition. **c** Long-term signal measurement using PIL EMG sensor. **d** Robust PIL NWs withstanding the wiping cleaning process after the first use. EMG sensors attached onto the musculocutaneous region of the human forearm (left), contaminated EMG sensors with dust and residues (middle), and clean EMG sensors after the wiping process (right). **e** Reusability of robust PIL NWs, depicting the negligible change in signal acquisition. **f** Change in SNR of EMG signals acquired by PIL sensors after the wiping process. **g** Change in skin impedance of the PIL sensors after the wiping process. Data in **f** represent means ± standard deviation (*n* = 3)

For wearable electronics designed to effectively acquire electrophysiological signals, the electrode surfaces must be exposed to direct contact with the human epidermis. Therefore, high mechanical robustness is required to endure external stress from direct contact with skin and to ensure reliable and long-term monitoring of electrophysiological signals. To address this need, we have patterned the biocompatible PIL AA NW electrode into a robust, open structure for electrophysiological sensors consisting of a set of three rectangular bar-shaped electrodes serving as the reference, ground, and measurement electrodes (Fig. 3a). The geometric configuration of these electrodes was specifically designed to optimize performance on the forearm (Fig. S15) [44–46]. We attached the as-prepared electrodes onto the human forearm to acquire reliable EMG signals when flexing, extending, and resting the human arm muscles (Fig. 3b). Moreover, the high robustness of the PIL AA NWs enabled us to conduct long-term EMG signal acquisition for three weeks (Fig. 3c). Specifically, the electrodes were used to measure EMG data at one-week intervals over a three-week period. The SNR values recorded at the end of the first, second, and third weeks were 40.46, 37.31, and 37.44, respectively. While a slight reduction was observed, the SNR remained sufficient for reliable EMG signal acquisition throughout the monitoring period. In addition, even after four months, the EMG sensor exhibited an SNR of 37.39, demonstrating its excellent long-term capability (Fig. S16). In case of contamination from foreign substances such as dust and residues during prolonged usage of the sensors, the used sensors can be cleaned through a wiping process while retaining their robustness (Fig. 3d). The sensors with bare and welded electrodes failed to obtain any signals after the cleaning process due to the delamination of NWs. However, after cleaning of the EMG sensors, only the PIL NW-based EMG sensor maintained its high signal quality on the same forearm (Fig. 3e). In addition, the acquired signals and signal-to-noise ratio (SNR) using the PIL sample showed negligible changes, with an SNR of 38.46 during the initial use and SNR of 36.59 and 38.42 during the subsequent two cycles of reuse (Figs. 3f and S17). The skin impedance of the PIL electrodes remained low to effectively monitor electrophysiological signals, with no significant differences observed before and after the cleaning tests (Fig. 3g). This characteristic suggests the potential of the LIPIL strategy for developing reusable wearable electronic devices.

### Practical Electrochemical Applications with Functionalization of NW Electrode

**Fig. 4:**
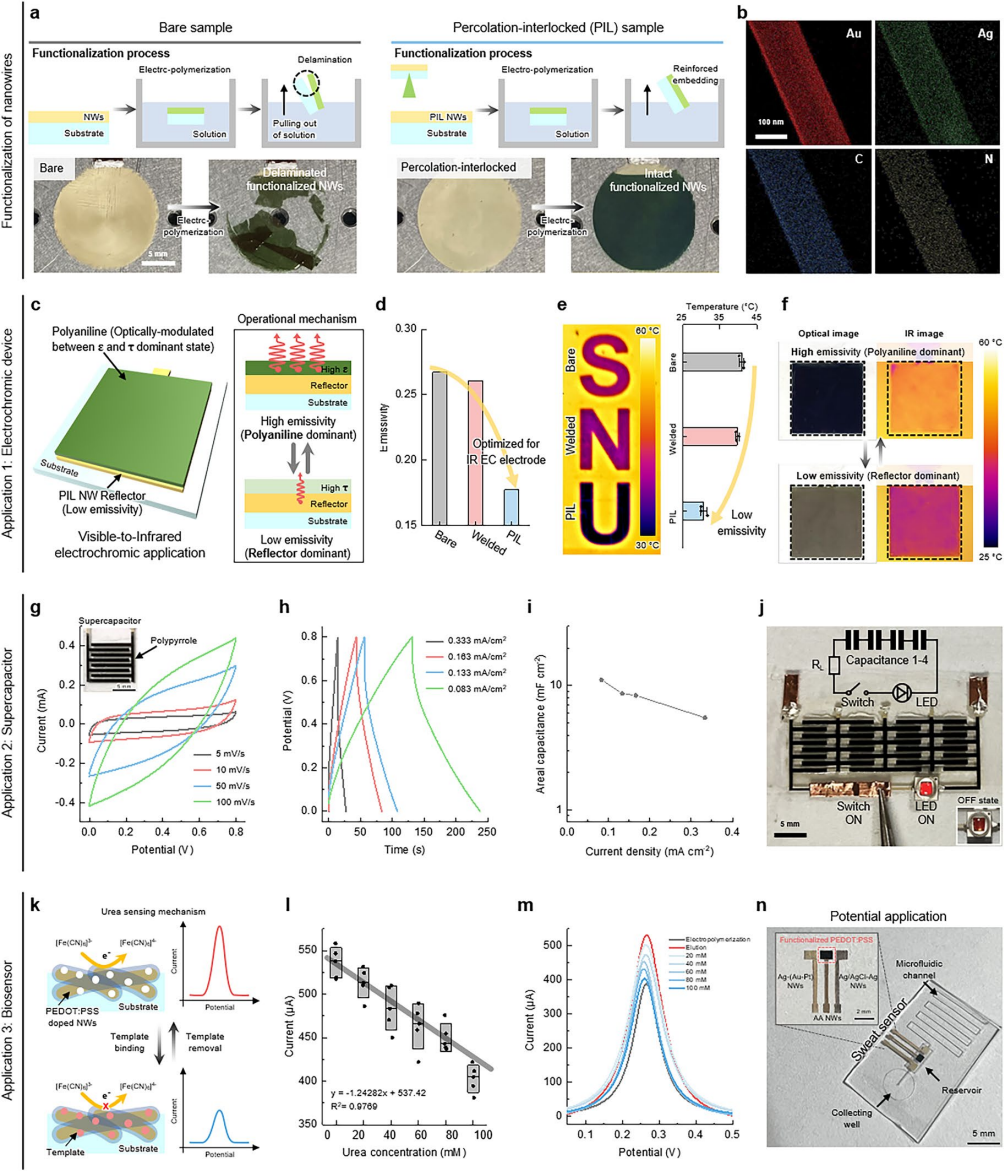
Functionalization of NWs and potential applications. **a** Schematic illustration of EP process, showing that the PIL NW-based electrode is highly suitable for functionalization. **b** TEM images of PANI-functionalized NWs, showing Au, Ag, C, and N atom components. **c** Schematic illustration of operational mechanism of the VIS-to-IR EC device based on PANI-functionalized NWs. **d** FTIR spectroscopy IR emissivity data of the NW samples. **e** Thermal image (left) and surface temperature (right) of the NW samples placed on the hot plate at 60 °C. **f** Digital images (left) and thermal images (right) of VIS-to-IR EC device, showing the high IR emissivity with the dark green appearance in the PANI-dominant state (top) and low IR emissivity with the light green appearance in the reflector-dominant state (bottom). **g** CV curves of the supercapacitor based on PPy-functionalized NWs. The inset shows an optical image of patterned PPy-functionalized NWs. **h** Galvanostatic charge/discharge curves of the supercapacitor. **i** Specific areal capacitance profile with varying applied current densities. **j** Supercapacitors connected in series illuminating a LED. **k** Schematic illustration of sensing mechanism of the PEDOT:PSS-functionalized NWs as a biosensor application. **l** Urea sensing performance of the biosensor. **m** DPV curves of the biosensor after EP (gray line), elution (red line), and urea adsorption (blue lines). **n** Potential application for sweat sensor integrated with microfluidic channel. The inset shows the magnified view of the sweat sensor. Data in **e** and **l** represent means ± standard deviation (*n* = 3 and *n* = 5, respectively)

EP is a strong technique to form CP layers on the surface of electrodes through an electrochemical process. The EP to enhance the functionality of NW electrodes also necessitates the direct exposure of electrodes to solution-based electrolytes for electrochemical reactions. This method involves the application of an electrical potential to the electrode immersed in solution-based electrolytes containing the monomers for polymerization to deposit CPs on the NW surface. However, NW networks are generally dispersed into the solution during long-time EP (Fig. S18 and Supplementary Video S3). Furthermore, although CPs were successfully deposited on the NW electrode, the functionalized NW electrode can still fail under even weak flow of the solution after EP (Fig. 4a). In this regard, the open-structured PIL AA NW electrode is highly suitable for solution-based EP due to its chemical stability and mechanical robustness on the substrate. After EP for PANI, the PIL NW electrode exhibits intact, functionalized NW networks coated with PANI. The energy-dispersive X-ray spectroscopy (EDX) results further exhibit the presence of carbon and nitrogen, confirming the successful formation of PANI on the surface of the AA NW (Fig. 4b and Supplementary Video S4). For diverse electrochemical devices with on-demand functionalities such as VIS-to-IR EC display, supercapacitor, and biosensor, we respectively deposited PANI, polypyrrole (PPy), and PEDOT:PSS onto PIL AA NW to provide new functionalities using EP process (Fig. S19).

First, we fabricated a VIS-to-IR EC device using a PANI-functionalized NW electrode, which modulates its optical properties depending on the applied voltage (Fig. 4c). Specifically, the EC device alternates between a PANI-dominant state and a reflector-dominant state with varying states of the PANI. In the PANI-dominant state, the device predominantly reflects the properties of PANI, exhibiting a visibly dark green appearance and high emissivity in the IR spectrum. Conversely, in the reflector-dominant state, the device displays a visibly light green appearance and low emissivity in the IR spectrum, influenced by the underlying reflector with high transparent PANI [47]. Furthermore, maximizing the optical difference in IR spectra between two distinct states for high IR device performance requires enhanced performance of the reflector by lowering its IR emissivity. In this regard, the PIL NW electrode exhibits superior IR performance as a reflector compared to bare and welded electrodes (Figs. 4d and S20). The thermal image of each electrode placed on the hot plate at 60 °C visually demonstrates that the PIL NW electrode achieves the lowest surface temperature, attributed to its lower emissivity (Fig. 4e). Benefiting from the improved optical properties, we have fabricated the EC device and assessed its performance across the VIS-to-IR spectrum. In the PANI-dominant state, the device displayed dark green coloration and relatively high surface temperature, while in the reflector-dominant state, it showed light green coloration and low surface temperature (Figs. 4f and S21).

Second, we devised a supercapacitor by functionalizing the PIL NWs patterned in a comb-drive design with PPy (Fig. S22). To examine capacitive performance, we conducted the cyclic voltammetry (CV) tests with various scanning rates of 5, 10, 50, and 100 mV s^−1^. The PPy-based supercapacitor demonstrates representative super-capacitive behavior, with the geometric structure retaining stability even at high scanning rates in CV sweeps, which is attributed to the chemical durability of PPy-functionalized NW electrodes (Fig. 4g). Additionally, the PPy-based supercapacitor exhibited linear charging-discharging profiles, which indicates a classical type of ideal capacitive performance (Fig. 4h). Based on the galvanostatic charge–discharge (GCD) curves, we calculated the specific areal capacitance with 5.51, 8.32, 8.63, and 11.09 mF cm^−2^ at 0.083, 0.133, 0.163, and 0.333 mA cm^−2^, respectively (Fig. 4i). Additionally, the supercapacitor exhibits a power density of 0.0332 mW cm^−2^ and energy density of 0.986 μWh cm^−2^ (Fig. S23). Moreover, connecting four PPy-based supercapacitor units in series allowed them to power a light-emitting diode (LED), demonstrating a practical application of this technology (Figs. 4j and S24).

Finally, we fabricated a biosensor based on a MIP using PEDOT:PSS deposited via a solution-based EP process, which specifically detects the concentration of urea in biofluids. The MIP-based biosensor is generally designed to selectively bind to specific molecules, forming a polymer matrix that mimics the shape and functional groups of target molecules [48]. During the EP process with CP monomers and target molecules, the target molecule is embedded within the polymer matrix. After polymerization, the removal of the target molecule leaves behind specific cavities within the matrix, allowing for selective binding of the target in subsequent applications. Under low concentrations, the electrochemical reaction between electrolyte and electrode actively occurs through the empty spaces, making the peak of the electrical signal high (Figs. 4k and S25). However, as the concentration of urea gets higher, the number of spaces for electrochemical reaction becomes sparse, making electrical signal peak smaller. The DPV with a 1 M potassium chloride (KCl, Sigma-Aldrich) solution containing 1 mM potassium ferricyanide (K_3_Fe[CN]_6_, Sigma-Aldrich) is employed to quantitatively analyze the concentration of urea in the solution. The DPV current peak decreases linearly as the concentration of urea increases linearly from 0 to 100 mM (Fig. 4l). The detailed current peak exhibits a distinct single peak of the redox agent (K_3_Fe[CN]_6_) in the range of 0–0.5 V (Fig. 4m). As expected, the current peaks are changed linearly depending on the number of electrochemical reaction sites between just after EP and elution states. Furthermore, for practical application in skin electronics, the MIP-based biosensor on a PET substrate can be integrated with a microfluidic device using plasma bonding (Fig. 4n). Utilizing the LIPIL method, which can be applied regardless of NW type, the PEDOT-functionalized MIP NW, Ag-AAP NW, and Ag–AgCl NW can be patterned to serve as the working electrode, counter electrode, and reference electrode, respectively. Using the microfluidic device equipped with the biosensor, sweat can be collected from the skin and directed through a collection well into a reservoir, where biomarkers in the sweat can then be analyzed by the integrated MIP-based sensor. By maintaining stable sensing performance even in direct contact with biofluids, the LIPIL method highlights its applicability in biofluid-rich environments, providing a critical advantage for the development of reliable MIP-based biosensors.

## Conclusions

In this paper, we proposed the LIPIL of the NW network by irradiating a CW laser from the backside of the transparent polymeric substrates to reinforce the open structure of NW networks. The newly developed LIPIL method tremendously reinforced the mechanical and electrical properties of the NW networks. Even though the NW networks with open structures were exposed to severe and repetitive friction, they maintained their properties, which is an important feature for reusable physiological applications that require direct contact with skin. Our PIL NW-based electrode demonstrated its capacity as a physiological EMG sensor, showing negligible performance loss after washing or wiping for reusable purposes. Furthermore, the PIL NW-based electrode can be functionalized variously by depositing various CPs onto the NW network with solution-based EP, thereby enabling desirable functions such as EC device, energy storage device, and biosensor. Lastly, the proposed LIPIL method is a powerful strategy since it can be applied to diverse NWs and polymeric substrates, which broadens the combinations of NWs and substrates for their on-demand applications. Therefore, we believe that our LIPIL method enhances the capability of NWs as key materials for soft electronics.

## Supplementary Information

Below is the link to the electronic supplementary material.

Supplementary file1 (DOCX 14754 KB)

Supplementary file2 (MP4 4247 KB)

Supplementary file3 (MP4 5064 KB)

Supplementary file4 (MP4 11222 KB)

Supplementary file5 (MP4 7163 KB)
