# Supplementary material for: Laser-Induced Nanowire Percolation Interlocking for Ultrarobust Soft Electronics
Source: Nanomicro Lett. 2025 Jan 31;17:127. doi: 10.1007/s40820-024-01627-7 (PMC11785881; doi:10.1007/s40820-024-01627-7)
Supplement: Supplementary file 1 — Supplementary file1 (DOCX 14754 KB) [file 40820_2024_1627_MOESM1_ESM.docx]

Supporting Information for

**Laser-Induced Nanowire Percolation Interlocking for Ultrarobust Soft Electronics**

Yeongju Jung^1,#^, Kyung Rok Pyun^1, #^, Sejong Yu^1^, Jiyong Ahn^1^, Jinsol Kim^1^, Jung Jae Park^1^, Min Jae Lee^1,2^, Byunghong Lee^2^, Daeyeon Won^1^, Junhyuk Bang^1^, Seung Hwan Ko^1,3^*

^1^ Applied Nano and Thermal Science Lab, Department of Mechanical Engineering, Seoul National University, 1 Gwanak-ro, Gwanak-gu, Seoul, 08826, South Korea

^2^ Energy Device Research Team, Hyundai Motor Company, 37 Cheoldobangmulgwan-ro, Uiwang-si, Gyeonggi-do, 16082, South Korea

^3^ Institute of Advanced Machinery and Design (SNU-IAMD), Seoul National University, Gwanak-ro, Gwanak-gu, Seoul, 08826, South Korea

^#^ Yeongju Jung and Kyung Rok Pyun contributed equally to this work.

*Corresponding author. E-mail: [maxko@snu.ac.kr](mailto:maxko@snu.ac.kr) (Seung Hwan Ko)

**S1 Superiority of LIPIL method**

***Superiority of LIPIL method in processing:*** Several approaches have been proposed to address the issue of weak adhesion, including conventional embedding methods, substrate surface engineering, chemical treatments, and other processes. However, conventional embedding methods, such as those using polyimide (PI) substrates with outstanding thermal and mechanical properties, often require the use of toxic solvents and high-temperature processes during substrate fabrication [S1]. These conditions can lead to the degradation of NWs, posing a significant limitation for their application. Substrate surface engineering, which involves modifying the roughness of the substrate surface, may introduce complexities in fabrication and is not ideal for soft electronics [S2]. Chemical treatments often fail to achieve strong adhesion because they primarily rely on van der Waals forces [S3]. Recently reported diffusion-induced methods also face some challenges such as the need for shadow masks for selective patterning of the electrode and their applicability being limited to specific metallic materials and substrates, thereby restricting their broader use [S4]. The LIPIL method presents significant advantages in overcoming the limitations of previous technologies.

***Versatility of materials:*** The versatility of the LIPIL method in accommodating a wide range of materials is crucial. The specific requirements of different applications necessitate diverse material properties. For instance, in the context of metallic NWs, the choice between Ag NW and Cu NW depends on whether high electrical conductivity or cost-effectiveness is the priority. Additionally, for applications requiring biocompatibility, AA NWs can be utilized, while Pt-containing AAP NWs are suitable for catalytic effects.

Furthermore, the method is adaptable to various substrate types depending on the required flexibility. For typical soft electronics, the LIPIL technique can be applied to PET substrates. In applications requiring enhanced stretchability, TPU substrates are preferable. Additionally, for scenarios demanding both high adhesion and stretchability, such as the reliable integration of multiple modules, SEBS, with its block copolymer properties, can be employed. The LIPIL method is also suitable for creating transparent electrodes that operate not only in the VIS spectrum but also in the IR range. This capability is based on the use of PE substrate which is known as VIS-to-IR transparent substrate. Traditional transparent electronics have primarily focused on visibility in the VIS range; however, there is an emerging need for transparency in the IR spectrum as well. The detailed explanation will be discussed in **S2**.

**S2 Potential of LIPIL method**

***Potential of LIPIL method for broadband transparent electrode in the visible-to-infrared range:*** As detailed in **S1**, the LIPIL method represents a versatile strategy capable of employing various polymer substrates and, notably, utilizing a PE substrate allows for the fabrication of broadband transparent electrodes. Recently, there has been significant advancement in developing transparent electrodes that extend beyond the VIS spectrum into the IR range. The need for IR-transparent electrodes primarily arises from their critical role in thermal management applications, which include regulating heat transfer in various systems. These electrodes often utilize metal-based deposition processes to dynamically control optical properties in the IR region, thereby enabling advanced solar and radiative thermal management applications [S5-S7]. However, traditional methods for fabricating VIS-to-IR transparent electrodes, such as photolithography, are often complex and not straightforward. The LIPIL method addresses these challenges by selectively patterning a percolation network grid on PE film, thus developing a broadband transparent electrode (refer to **Fig. S4** for the details). This approach simplifies the fabrication process and enhances the efficiency of producing IR-transparent electrodes, making it a promising technique for future applications in thermal management and other advanced technologies.

**S3 Optimization of the NW-based electrode fabrication and laser processing**

Fabricating the NW electrodes with outstanding mechanical and electrical features is significantly important for their stable operation. In this study, the NW-based electrode is produced by vacuum filtration of the NW solutions, and the volume of the NW solution is an important factor that determines the electrical properties of NW-based electrode. Note that we standardized the concentration of each NW solution by carefully adjusting the volume of ethanol, ensuring minimal variation between the solutions. The electrical resistance of the NW network was subsequently controlled by varying the volume of the applied NW solution. Furthermore, since we employed a 532 nm wavelength laser to enhance the mechanical and electrical properties of the NW electrodes, the optical properties depending on the volume of the NWs also play an important role in the effectiveness of the laser processing. Given that the surface plasmonic effect of the NWs results in high laser absorption in the VIS wavelength region, determining the optimal volume of NWs is essential for optimizing laser processing.

We measured the UV-Vis transmittance and absorption of the NW-based electrodes for various NW solution volumes (200, 400, 600, 800 and 1000 µL) (**Fig. S5**). NW electrodes with a smaller volume of solution show high transparency, indicating that they are not highly absorptive of our VIS laser (532 nm wavelength) to effective photothermal effect on the surface of the NW electrode. In contrast, a large amounts of NW networks display significant VIS light absorption.

Based on the optical properties of the prepared NW electrodes, we investigated the laser welding effect on the electrical properties using FSL and BSL irradiation with varying laser parameters (**Fig. S6**). We confirmed that increasing the NW volume enhances the welding effect and consequently improves the electrical properties. For FSL irradiation, 100 mW was found to be the optimal laser power condition, while for BSL irradiation, 300 mW was identified as optimal. To simplify the experimental process, we established these conditions as fixed parameters for electrode fabrication.

**S4 Simulation**

We performed a theoretical study through the heat transfer module in COMSOL Multiphysics to investigate the photothermal effects of NW electrodes under FSL and BSL irradiation. The simulation parameters were based on experimental data, and complex structures, such as NW percolation, were simplified for computational efficiency.

***Heat transfer simulations:*** Based on the features of thick NW electrode, as mentioned above, to analyze the heat transfer mechanisms, we initially prepared the AA NW electrode sample with a thickness of 20 µm transferred onto a 100 µm thick PE substrate. To mitigate potential deformation of the PE substrate, we utilized a supplementary glass substrate, which is positioned beneath the PE substrate during frontside laser irradiation and placed on top of the AA NW layer for backside laser irradiation. Subsequently, to theoretically determine the temperature distribution, we conducted heat transfer simulations incorporating a laser-induced moving heat flux modeled with a 2D Gaussian distribution (**Eq. S1**).

$Heat flux\left( x,y \right)=A\left[ \frac{P}{2\pi\sigma_{x}\sigma_{y}}exp(-\left( \frac{\left( x-\mu_{x}\left( t \right) \right)^{2}}{2{\sigma_{x}}^{2}}+\frac{\left( x-\mu_{y}\left( t \right) \right)^{2}}{2{\sigma_{y}}^{2}} \right)) \right]$ (**S1**)

Here, $A$ denotes the absorptance of AA NWs, and $P$ represents the laser power of 300 mW. The center of the laser beam is characterized by $\mu_{x}\left( t \right)$ = 0 and $\mu_{y}\left( t \right)$ = $scan speed \times time$, where the scan speed is set to 100 mm s^-1^. The standard deviations, $\sigma_{x}$ and $\sigma_{y}$, are determined by the effective range of the laser beam.

Although the laser-induced thermal interaction cannot directly elucidate the percolation interlocking behavior, we examined the heat transfer mechanism as an indirect approach to understand the embedding mechanism of NWs because localized temperature profiles by laser irradiation can offer direct information about the substrate melting (**Fig. S7**). Under the FSL irradiation onto the NW surface, the temperature of NW surface significantly rises. However, the photothermal energy absorbed at the NW surface does not transfer effectively to the interface between NWs and substrate due to the thick NW-air composite structures. In contrast, the BSL irradiation allows the dense NW networks to absorb the substantial photothermal energy enough to cause the substrate melting. Therefore, the backside laser irradiation method provides the optimal conditions for the percolation interlocking of NW networks. Specifically, during the backside laser process, it is obvious that the interface (denoted as Point 1 in the **Fig. S8**) attained a maximum temperature corresponding to the melting point of PE substrate (approximately 110 °C) [S8], in contrast to the other regions that did not approach the melting point. Moreover, examining the temperature distributions in the z-axis directions, it was observed that the boundary region between the NW layer and the PE substrate exhibited temperatures exceeding the melting point of the polymer substrate. In conclusion, the simulation results explained that the thermal energy absorbed at the interface can cause the substrate to momentarily melt, thereby potentially interlocking the NW percolation network within the polymer substrate.

**S5** **Optimization of Resolution and Mechanical Robustness in PIL NW Electrodes**

We presented the optimized conditions for PIL NW electrodes in **Fig. 2b**, using a laser power of 300 mW and a scanning speed of 100 mm s^-1^. However, increasing the scanning speed allows for the fabrication of electrodes with higher resolution at the expense of some mechanical robustness (**Fig. S9a**). This indicates a clear trade-off between mechanical robustness and resolution, which is a critical aspect in the fabrication of PIL NW electrodes using laser processing.

To address this trade-off, we investigated the electrical resistance of the patterned PIL NW electrodes after the wiping process under varying laser scanning speeds, focusing on the resulting pattern widths and electrical resistance (**Fig. S9b**). Increasing the laser scanning speed significantly enhances the resolution of the patterned electrodes, yielding narrower pattern widths. However, the mechanical robustness of the electrodes, as indicated by their electrical resistance after the wiping process, decreases at higher scanning speeds. Specifically, the PIL NW samples prepared at a scanning speed of 500 mm s^-1^ exhibit no electrical conductivity, highlighting the limitation of extremely high-speed laser processing. As a result, we concluded that electrodes with widths as narrow as approximately 30 μm can maintain electrical resistance within a usable range for practical applications, establishing a minimum resolution while preserving its functionality. Based on our analysis, a scanning speed of approximately 200 mm s^-1^ at a fixed laser power of 300 mW provides an optimal balance between pattern resolution and mechanical robustness, showing manageable variability in both pattern width and electrical resistance.

However, the LIPIL method allows for multiple cycles of laser irradiation, which enhances both the mechanical robustness and electrical resistance of the electrodes (**Fig. 2e**). This approach provides a practical means to compensate for the trade-offs associated with higher resolution, ensuring that the electrodes meet the demands of specific applications. By combining appropriate scanning speeds, multiple laser irradiation cycles, and a clear understanding of application-specific priorities, the trade-off between resolution and mechanical robustness can be effectively mitigated.

**Supplementary Figures and Table**


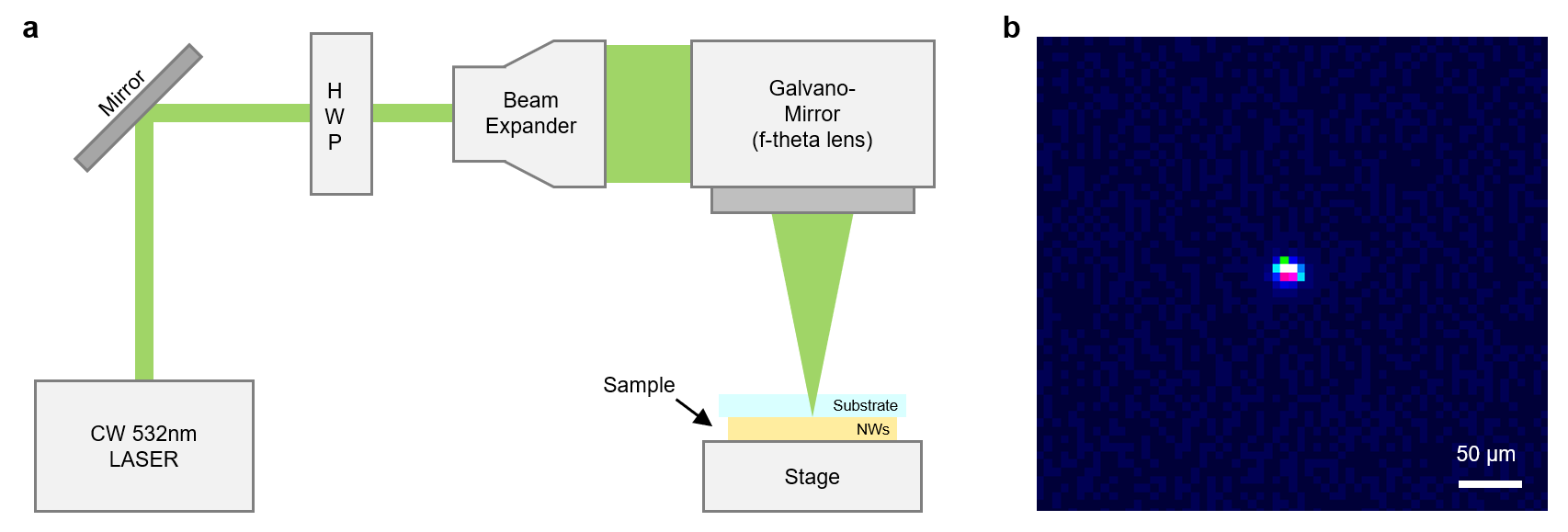


**Fig. S1** Operational configuration for LIPIL. **(a)** Schematic illustration for laser experimental setup. An optical system is designed for precise laser beam control, utilizing a continuous-wave (CW) laser with a wavelength of 532 nm. The system includes a mirror and half-wave plate (HWP) to adjust the direction of beam, followed by a beam expander to enhance beam quality and enable precise focusing. The expanded beam is directed by a galvano mirror, and an f-theta lens ensures uniform focus on a planar surface. The laser interacts with a sample placed on a substrate, which is mounted on a stage for precise positioning. **(b)** Laser beam profile with diameter approximately 17.55 μm captured at the focal point.


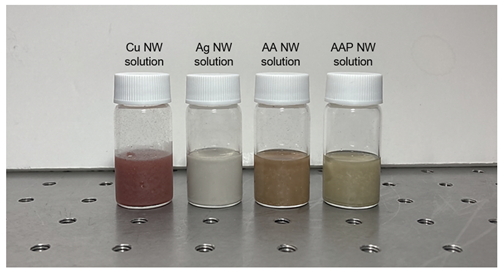


**Fig. S2** Metallic NW solutions such as Cu NW, Ag NW, AA NW, and AAP NW solution

**
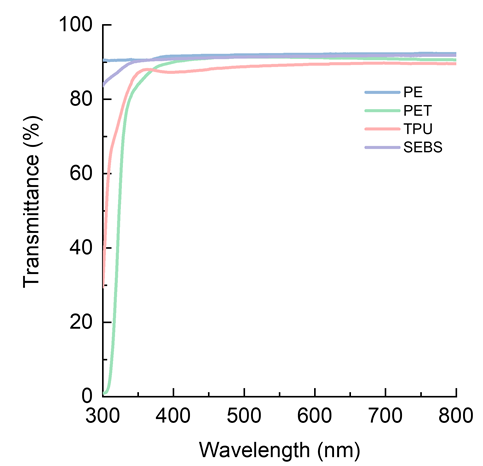
**

**Fig. S3** UV-VIS spectroscopy transmittance data of various transparent substrates.

**
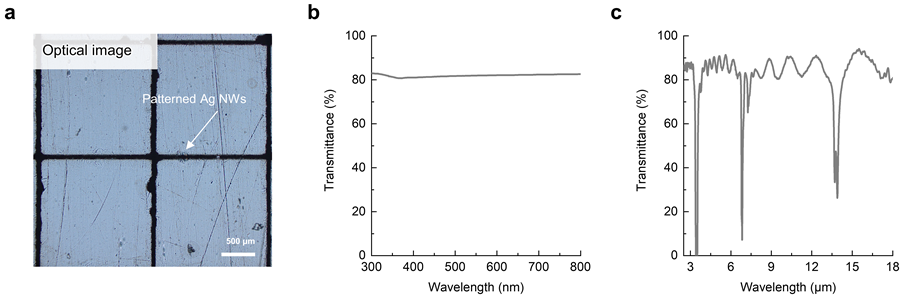
**

**Fig. S4** LIPIL Ag NWs grid on PE film for broadband transparent conductive electrode in the VIS-to-IR range. **(a)** Optical image of grid-patterned Ag NWs on PE film by LIPIL method. **(b)** VIS transmittance data of the electrode. **(c)** IR transmittance data of the electrode.

**
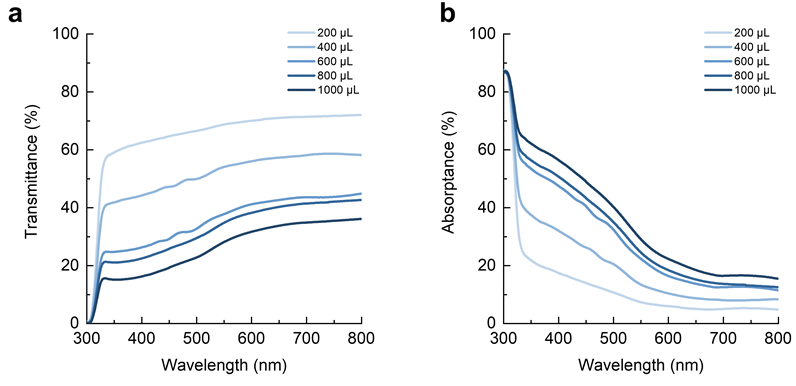
**

**Fig. S5** UV-VIS spectroscopy data of AA NWs transferred on PET substrate with varying NW solution volumes. **(a)** Transmittance data. **(b)** Absorption data.


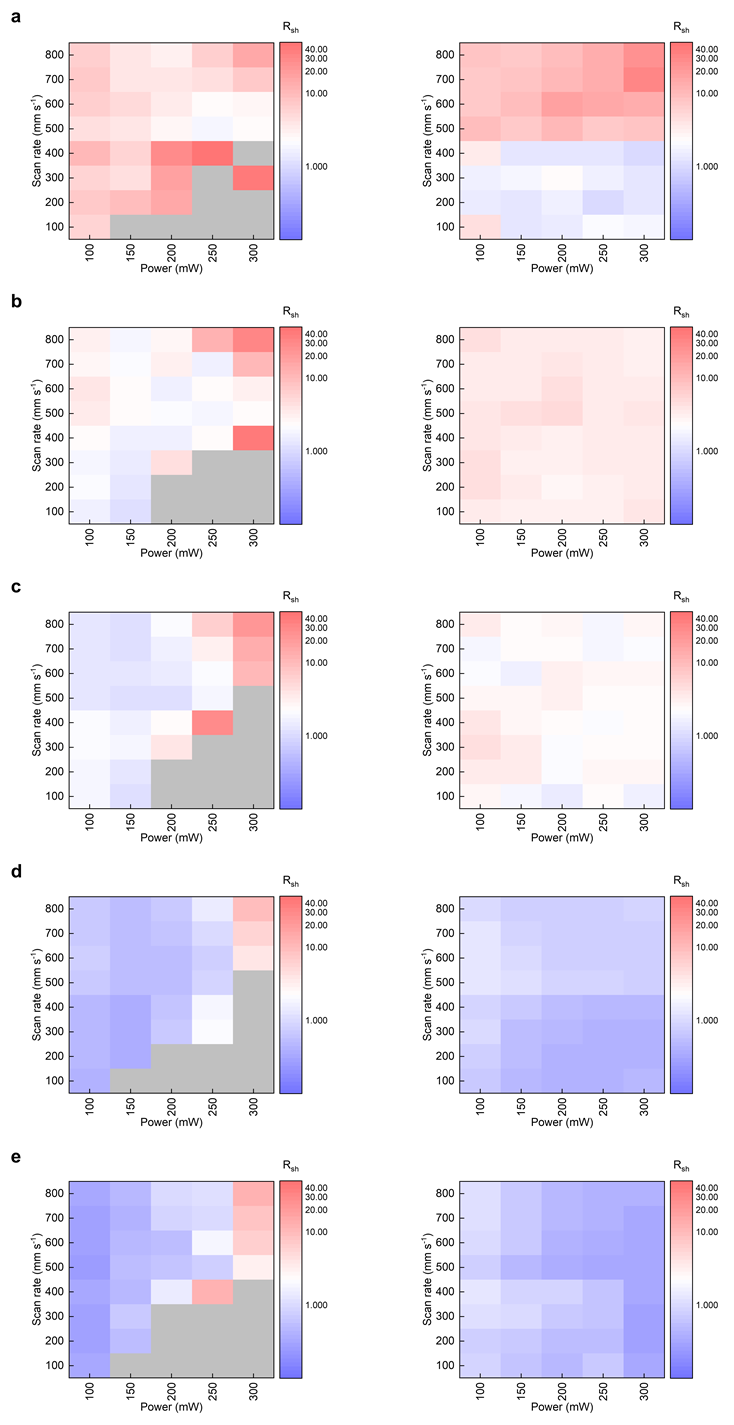


**Fig. S6** Sheet resistance of welded (left column profiles) and PIL NW electrodes (right column profiles) at varying laser processing conditions with different NW solution volumes. **(a)** 200 μL. **(b)** 400 μL. **(c)** 600 μL. **(d)** 800 μL. **(e)** 1000 μL. The gray colors indicate the substrate failure

**
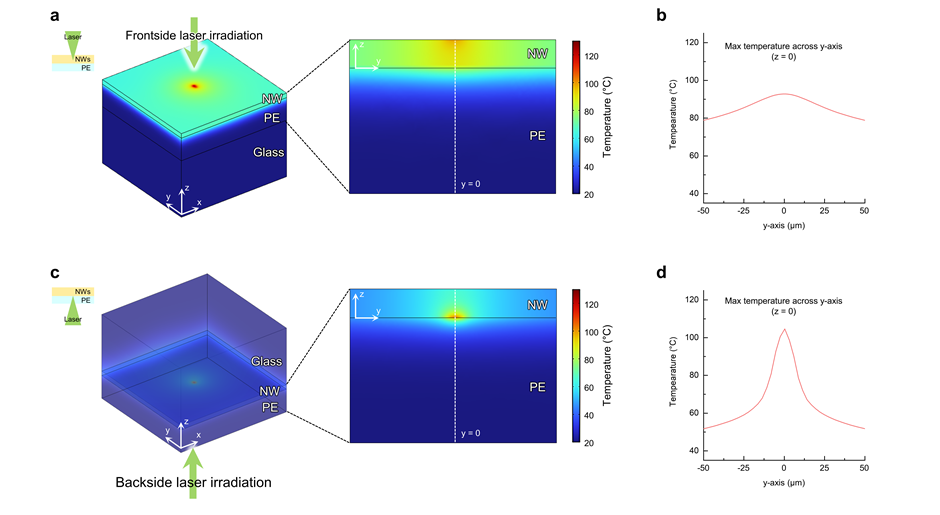
**

**Fig. S7** Heat transfer simulation analysis according to laser irradiation direction. **(a)** COMSOL image of FSL irradiation. **(b)** Maximum temperature distribution across y-axis (z = 0, the interface between NW layer and PE substrate). **(c)** COMSOL image of BSL irradiation. **(d)** Maximum temperature distribution across y-axis (z = 0, the interface between NW layer and PE substrate).

**
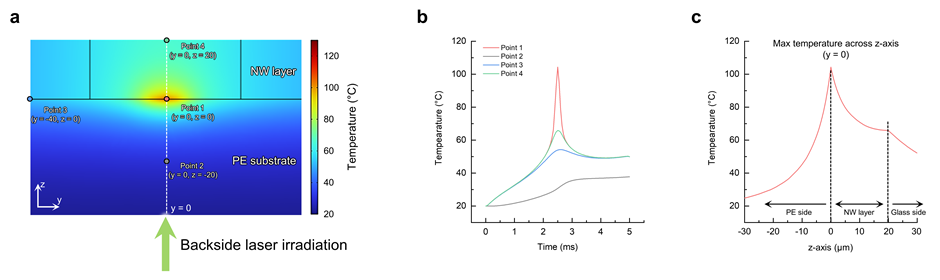
**

**Fig. S8** Heat transfer simulation of LIPIL for quantitative evaluation of temperature distribution. **(a)** COMSOL image showing the heat transfer results when a laser is irradiated from backside of the PE substrate. **(b)** Temperature distribution over time at 4 points. **(c)** Maximum temperature distribution across z-axis (y = 0).


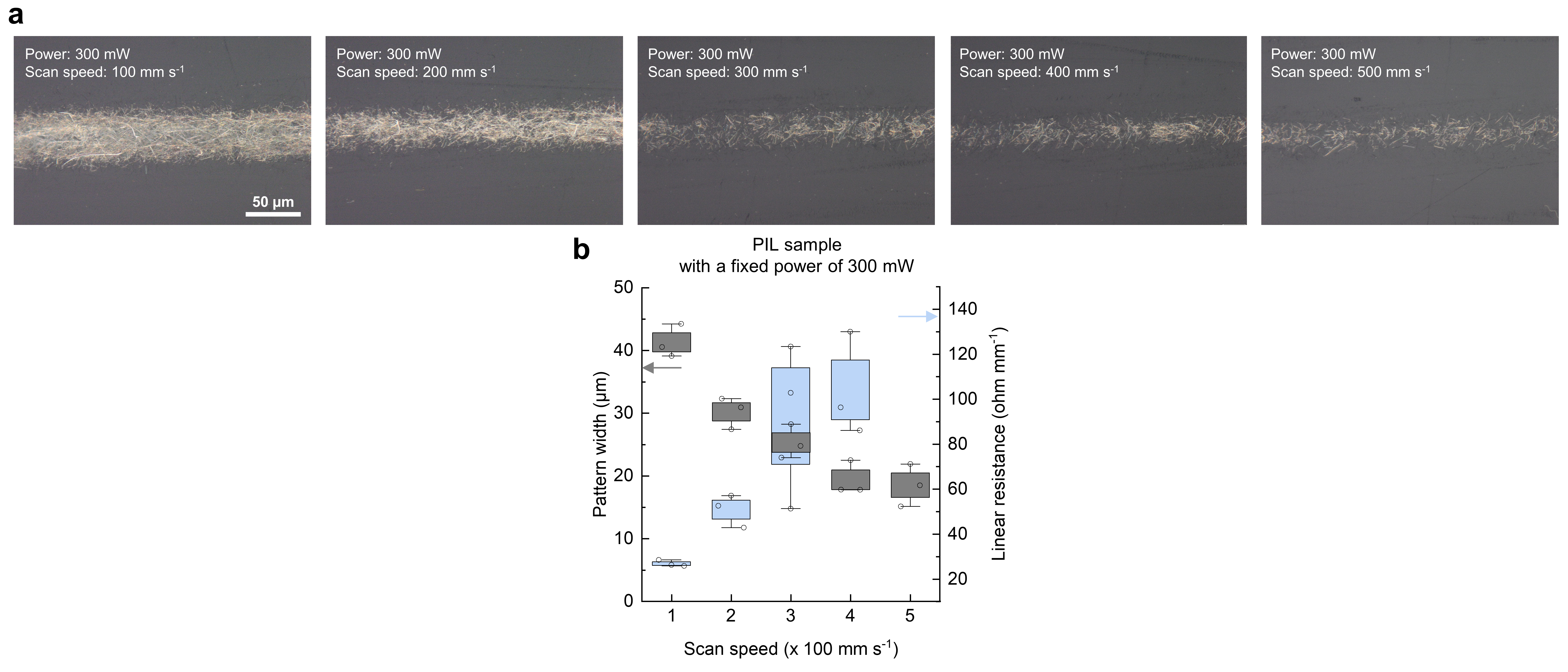


**Fig. S9** Width resolution of patterned PIL NWs at a fixed laser power of 300 mW. **(a)** Optical images of patterned PIL NW after wiping process with varying scan speeds of 100, 200, 300, 400, and 500 mm s^-1^. **(b)** a graph showing width and electrical resistance of each patterned PIL NW electrode. The PIL sample prepared with a scan speed of 500 mm s^-1^ shows no electrical conductivity


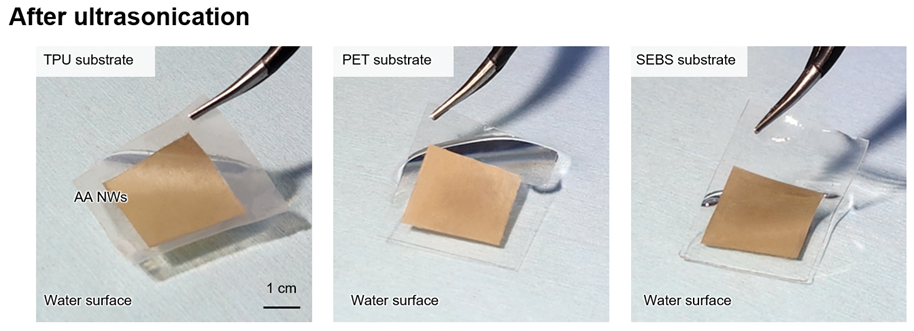


**Fig. S10** Strong adhesion of AA NWs on various substrates after ultrasonication


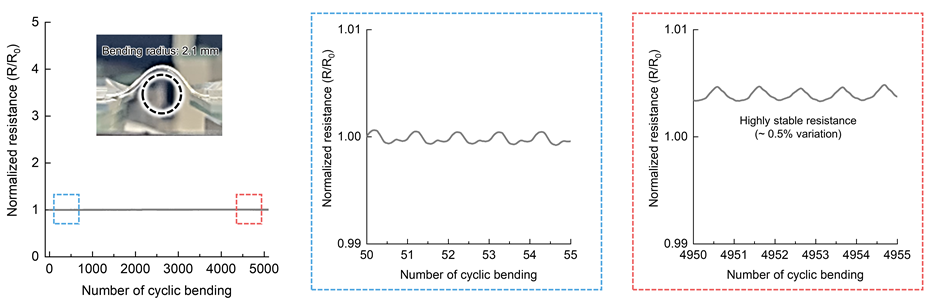


**Fig. S11** Normalized resistance changes of the PIL samples subjected to repeated bending cycles with a bending radius of 2.1 mm


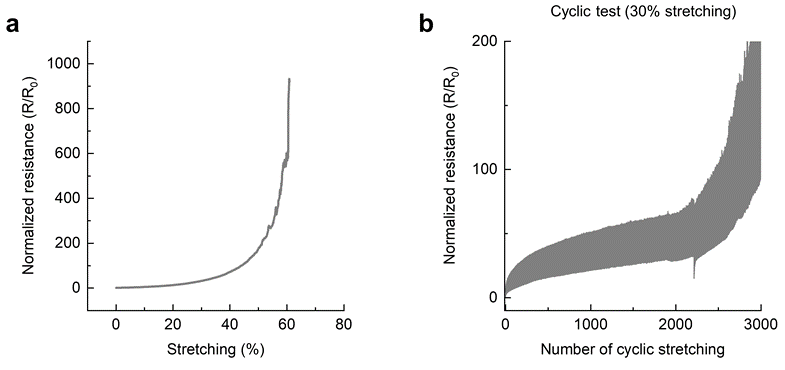


**Fig. S12** Normalized resistance changes of the PIL samples subjected to stretching tests. **(a)** Maximum stretching test. **(b)** Repeated stretching test with 30% stretching


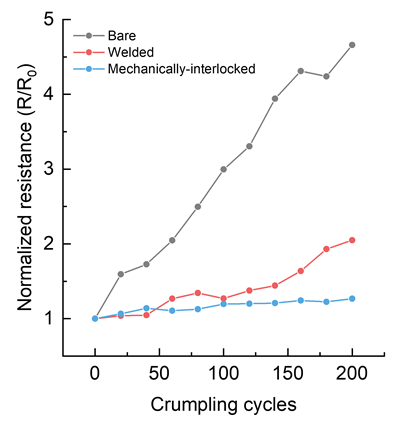


**Fig. S13** Normalized resistance changes of the bare, welded, and PIL samples subjected to crumpling

**
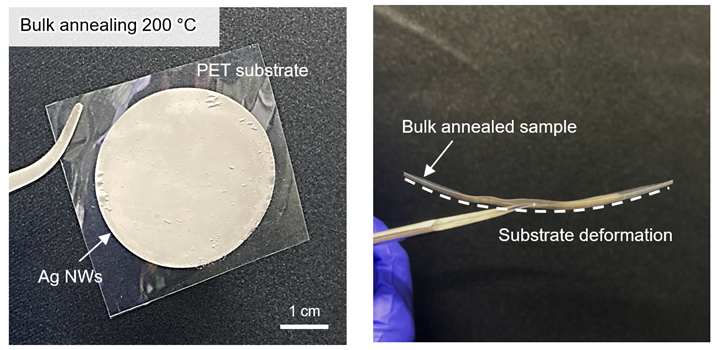
**

**Fig. S14** Plastic deformation of PET substrate on which Ag NWs are transferred after bulk annealing


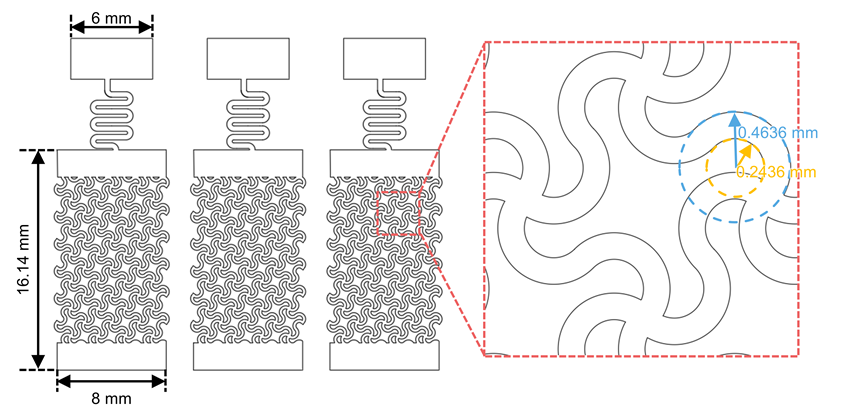


**Fig. S15** Design of electrophysiological sensor for acquiring EMG signals

**
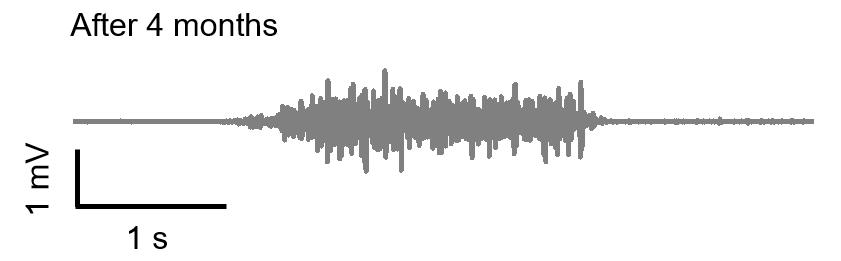
**

**Fig. S16** EMG signals measured after 4 months of sensor fabrication

**
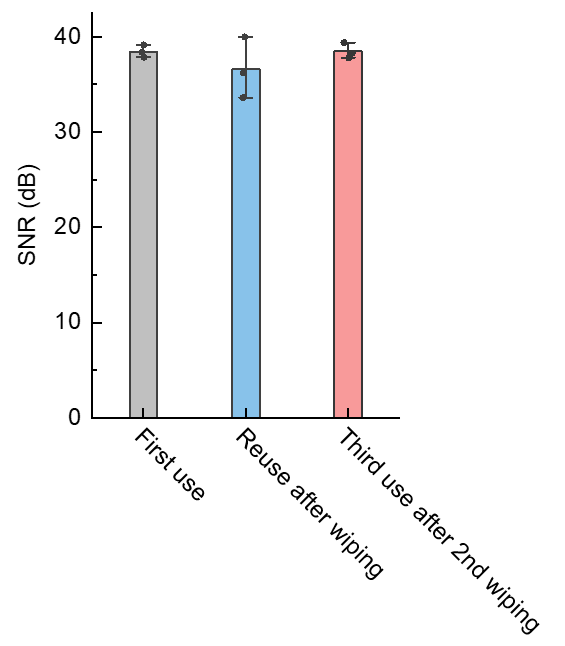
**

**Fig. S17** Change in SNR of EMG signals after repeated wiping processes for reuse


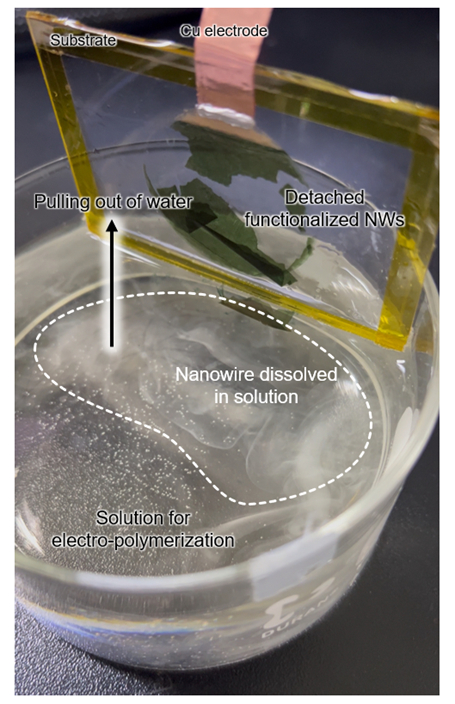


**Fig. S18** Delamination and dispersion issue of the bare NWs when they are withdrawn from water after the EP process


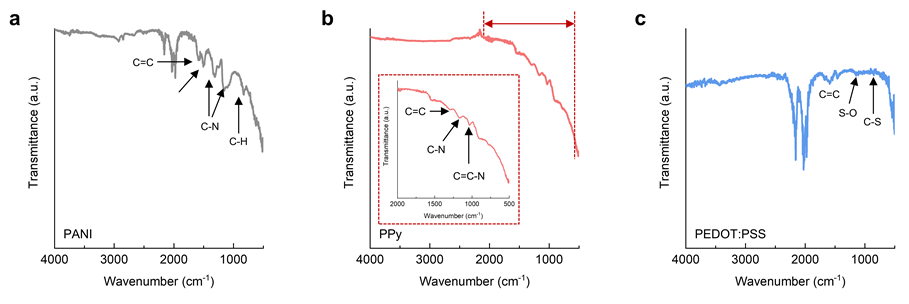


**Fig. S19** FTIR spectra of diverse CPs which are electropolymerized onto AA NWs. **(a)** PANI. **(b)** PPy. **(c)** PEDOT:PSS


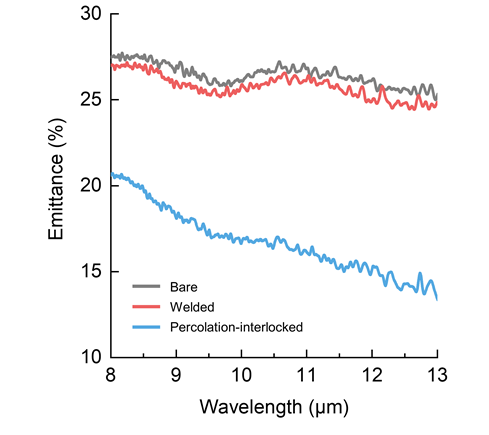


**Fig. S20** FTIR emittance data of the bare, welded, and PIL NW electrodes based on the PE substrates

**
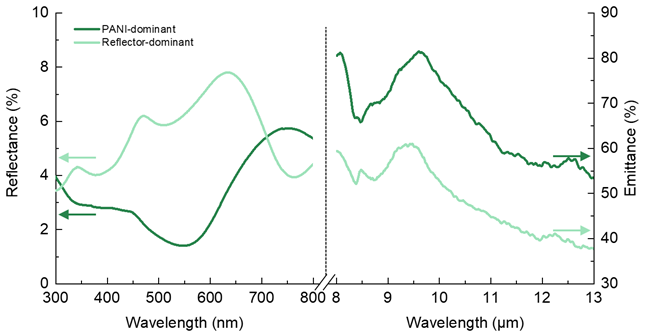
**

**Fig. S21** Optical properties of the PANI-functionalized PIL NWs at different states. The dark green and light green lines represent PANI-dominant state (high emissivity) and reflector-dominant state (low emissivity), respectively


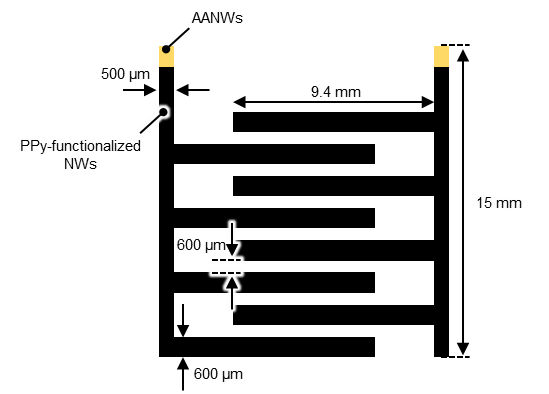


**Fig. S22** Design of the PPy-functionalized of PIL NWs as supercapacitor


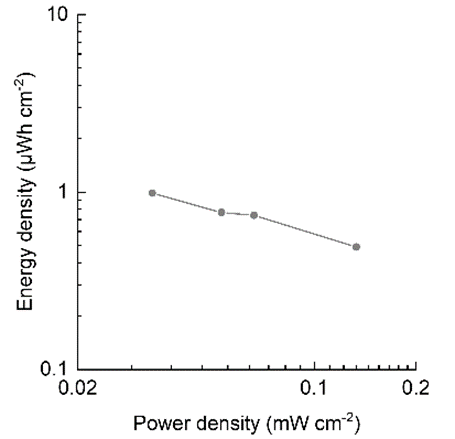


**Fig. S23** Energy and power density profiles of the PPy-functionalized NW-based supercapacitor


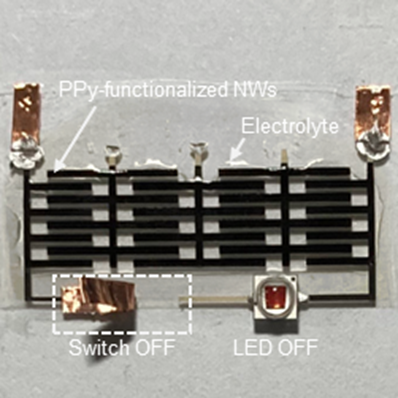


**Fig. S24** A series of supercapacitors connected to an LED in the switched-off state


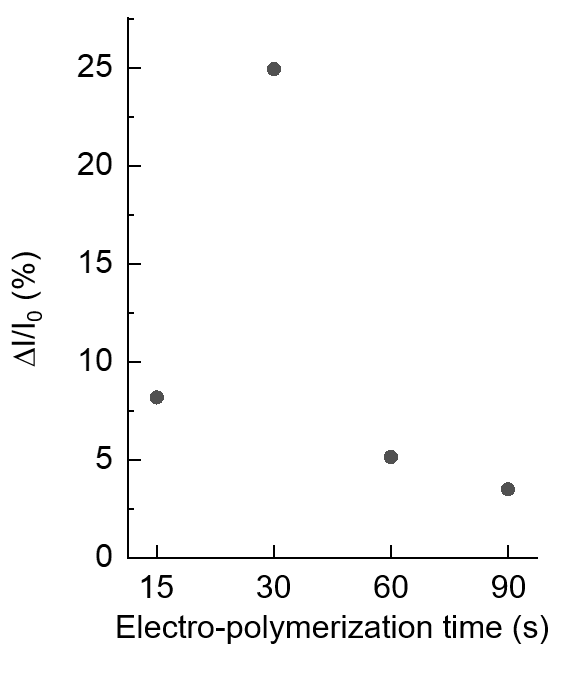


**Fig. S25** The sensitivity of MIP-based biosensor depending on the polymerization charge time at a constant potential of 1 V

**Table S1** Comparison of electrical resistance between NW electrodes fabricated by LIPIL process and other NW electrodes

| **Refs.** | **Types of materials** | **Sheet resistance (ohm sq^-1^)** | **Electrode density (mg cm^-2^)** | **Fabrication process** |  |
| --- | --- | --- | --- | --- | --- |
| [S9] | Cu NWs, rGO | 0.808 | 5 | Vacuum filtration |  |
| [S10] | Ag NWs | 8.6 | N/R | Annealing |  |
| [S11] | Ag NWs, ZnO | 13 | N/R | Annealing |  |
| [S12] | Cu NWs, TiO_2_ | 13 | N/R | Annealing |  |
| [S13] | Cu NWs | 17 | N/R | Spray coating |  |
| [S14] | Ag NWs | 29.63 | N/R | Vacuum filtration, Annealing |  |
| [S15] | Cu NWs | 30 | 0.6 | Bar coating |  |
| [S16] | Ag-Au NWs | 42.3 – 167.7 | 0.52 – 2.68 | Vacuum filtration |  |
| [S17] | Au NWs | 49 | N/R | Electrodeposition |  |
| [S18] | Au NWs | 400 | N/R | Homogenous coating using stabilizer |  |
| **This work** | **Ag-Au NWs** | **0.313** | **1.69** | **Backside laser irradiation (BSL)** |  |

N/R: Not reported

**Supplementary References**

1. K.K. Kim, J. Choi, J.-H. Kim, S. Nam, S.H. Ko, Evolvable skin electronics by *in situ* and in operando adaptation. Adv. Funct. Mater. **32**, 2106329 (2022). <https://doi.org/10.1002/adfm.202106329>
2. M. Ganji, L. Hossain, A. Tanaka, M. Thunemann, E. Halgren et al., Monolithic and scalable Au nanorod substrates improve PEDOT-metal adhesion and stability in neural electrodes. Adv. Healthc. Mater. **7**, e1800923 (2018). <https://doi.org/10.1002/adhm.201800923>
3. S. Ji, X. Chen, Enhancing the interfacial binding strength between modular stretchable electronic components. Natl. Sci. Rev. **10**, nwac172 (2022). <https://doi.org/10.1093/nsr/nwac172>
4. J. Cao, X. Liu, J. Qiu, Z. Yue, Y. Li et al., Anti-friction gold-based stretchable electronics enabled by interfacial diffusion-induced cohesion. Nat. Commun. **15**, 1116 (2024). <https://doi.org/10.1038/s41467-024-45393-x>
5. Y. Rao, J. Dai, C. Sui, Y.-T. Lai, Z. Li et al., Ultra-wideband transparent conductive electrode for electrochromic synergistic solar and radiative heat management. ACS Energy Lett. **6**, 3906–3915 (2021). <https://doi.org/10.1021/acsenergylett.1c01486>
6. M. Li, D. Liu, H. Cheng, L. Peng, M. Zu, Manipulating metals for adaptive thermal camouflage. Sci. Adv. **6**, eaba3494 (2020). <https://doi.org/10.1126/sciadv.aba3494>
7. C. Sui, J. Pu, T.-H. Chen, J. Liang, Y.-T. Lai et al., Dynamic electrochromism for all-season radiative thermoregulation. Nat. Sustain. **6**, 428–437 (2023). <https://doi.org/10.1038/s41893-022-01023-2>
8. Y. Na, S. Dai, C. Chen, Direct synthesis of polar-functionalized linear low-density polyethylene (LLDPE) and low-density polyethylene (LDPE). Macromolecules **51**, 4040–4048 (2018). <https://doi.org/10.1021/acs.macromol.8b00467>
9. W. Zhang, Z. Yin, A. Chun, J. Yoo, Y.S. Kim et al., Bridging oriented copper nanowire–graphene composites for solution-processable, annealing-free, and air-stable flexible electrodes. ACS Appl. Mater. Interfaces **8**, 1733–1741 (2016). <https://doi.org/10.1021/acsami.5b09337>
10. T. Tokuno, M. Nogi, M. Karakawa, J. Jiu, T.T. Nge et al., Fabrication of silver nanowire transparent electrodes at room temperature. Nano Res. **4**, 1215–1222 (2011). <https://doi.org/10.1007/s12274-011-0172-3>
11. M. Song, J.H. Park, C.S. Kim, D.-H. Kim, Y.-C. Kang et al., Highly flexible and transparent conducting silver nanowire/ZnO composite film for organic solar cells. Nano Res. **7**, 1370–1379 (2014). <https://doi.org/10.1007/s12274-014-0502-3>
12. H. Zhai, Y. Li, L. Chen, X. Wang, L. Shi et al., Copper nanowire-TiO_2_-polyacrylate composite electrodes with high conductivity and smoothness for flexible polymer solar cells. Nano Res. **11**, 1895–1904 (2018). <https://doi.org/10.1007/s12274-017-1807-9>
13. H. Zhai, R. Wang, X. Wang, Y. Cheng, L. Shi et al., Transparent heaters based on highly stable Cu nanowire films. Nano Res. **9**, 3924–3936 (2016). <https://doi.org/10.1007/s12274-016-1261-0>
14. J. Lee, P. Lee, H. Lee, D. Lee, S.S. Lee et al., Very long Ag nanowire synthesis and its application in a highly transparent, conductive and flexible metal electrode touch panel. Nanoscale **4**, 6408–6414 (2012). <https://doi.org/10.1039/c2nr31254a>
15. A.R. Rathmell, B.J. Wiley, The synthesis and coating of long, thin copper nanowires to make flexible, transparent conducting films on plastic substrates. Adv. Mater. **23**, 4798–4803 (2011). <https://doi.org/10.1002/adma.201102284>
16. H. Lee, S. Hong, J. Lee, Y.D. Suh, J. Kwon et al., Highly stretchable and transparent supercapacitor by Ag–Au core–shell nanowire network with high electrochemical stability. ACS Appl. Mater. Interfaces **8**, 15449–15458 (2016). <https://doi.org/10.1021/acsami.6b04364>
17. P.E. Lyons, S. De, J. Elias, M. Schamel, L. Philippe et al., High-performance transparent conductors from networks of gold nanowires. J. Phys. Chem. Lett. **2**, 3058–3062 (2011). <https://doi.org/10.1021/jz201401e>
18. A. Sánchez-Iglesias, B. Rivas-Murias, M. Grzelczak, J. Pérez-Juste, L.M. Liz-Marzán et al., Highly transparent and conductive films of densely aligned ultrathin Au nanowire monolayers. Nano Lett. **12**, 6066–6070 (2012). <https://doi.org/10.1021/nl3021522>
